# ELECTRONIC USE AND SLEEP, SURPRISING BEDFELLOWS: RESTFULNESS EFFECTS OF ELECTRONICS USE PRIOR TO BEDTIME FOR THE 40-PLUS

**DOI:** 10.1093/geroni/igz038.1936

**Published:** 2019-11-08

**Authors:** G Rainville, Cheryl Lampkin

**Affiliations:** AARP, Washington, District of Columbia, United States

## Abstract

Getting restful sleep is essential to well-being but stress and poor sleep habits may make sleeping through the night challenging. This research explored life event stressors and pre-sleep activities among 2,464 randomly selected Americans age 40 and older (using Ipsos’ KnowledgeNetwork panel) to determine their joint effects on mental well-being. Respondents reported how often they engaged in twelve individual behaviors within an hour of going to sleep. These behaviors (found to be inter-correlated) were combined using EFA into four factors representing levels of engagement in each of four classes of pre-sleep activities: pre-sleep electronics use (e.g. texting/e-mail before bed), deep relaxation activities, reliance on sleep-aids, and “nightowl” behaviors (i.e., snacking). Counter to expectations, only electronics use had significant conditional effects on the path between a life events stressor index (a count of current, potentially stressful life events) and scores on the positively-framed Warwick Edinburgh well-being scale (WEMWBS). How often one sleeps through the night also had unexpected effects in a conditional path analysis. A somewhat-involved relationship emerges between each of the theoretically-relevant measures. First, the negative impact of stress is moderated by sleeping through the night. Sleeping through the night is, counter to previous studies on electronics use and sleep, mediated by the use of electronics prior to sleep. We propose that mechanisms (such as the nature of backlighting used in electronics) that hamper restfulness may be offset by relaxation effects or by setting one’s ducks in a row by texting/emailing before going to sleep.

